# Spermatogenesis-Specific Features of the Meiotic Program in *Caenorhabditis elegans*

**DOI:** 10.1371/journal.pgen.1000611

**Published:** 2009-08-21

**Authors:** Diane C. Shakes, Jui-ching Wu, Penny L. Sadler, Kristen LaPrade, Landon L. Moore, Alana Noritake, Diana S. Chu

**Affiliations:** 1 Department of Biology, College of William and Mary, Williamsburg, Virginia, United States of America; 2 Department of Biology, San Francisco State University, San Francisco, California, United States of America; 3 Department of Biology, University of Southern Indiana, Evansville, Indiana, United States of America; Lawrence Berkeley National Laboratory and University of California Berkeley, United States of America

## Abstract

In most sexually reproducing organisms, the fundamental process of meiosis is implemented concurrently with two differentiation programs that occur at different rates and generate distinct cell types, sperm and oocytes. However, little is known about how the meiotic program is influenced by such contrasting developmental programs. Here we present a detailed timeline of late meiotic prophase during spermatogenesis in *Caenorhabditis elegans* using cytological and molecular landmarks to interrelate changes in chromosome dynamics with germ cell cellularization, spindle formation, and cell cycle transitions. This analysis expands our understanding *C. elegans* spermatogenesis, as it identifies multiple spermatogenesis-specific features of the meiotic program and provides a framework for comparative studies. Post-pachytene chromatin of spermatocytes is distinct from that of oocytes in both composition and morphology. Strikingly, *C. elegans* spermatogenesis includes a previously undescribed karyosome stage, a common but poorly understood feature of meiosis in many organisms. We find that karyosome formation, in which chromosomes form a constricted mass within an intact nuclear envelope, follows desynapsis, involves a global down-regulation of transcription, and may support the sequential activation of multiple kinases that prepare spermatocytes for meiotic divisions. In spermatocytes, the presence of centrioles alters both the relative timing of meiotic spindle assembly and its ultimate structure. These microtubule differences are accompanied by differences in kinetochores, which connect microtubules to chromosomes. The sperm-specific features of meiosis revealed here illuminate how the underlying molecular machinery required for meiosis is differentially regulated in each sex.

## Author Summary

Sperm and oocytes contribute equal but unique complements of DNA to each new life. Both types of cells arise from meiosis, a multi-step program during which chromosomes replicate, pair and recombine, then divide to generate haploid gametes. Simultaneously, each cell type also differentiates via distinct developmental programs. Spermatogenesis rapidly produces many small, motile sperm with highly protected chromatin, while oogenesis occurs at a slower rate to yield fewer large, immobile, nutrient-rich oocytes. We provide a detailed molecular analysis of key landmark events of spermatogenesis and identify spermatogenesis-specific features of meiosis in the model organism *C. elegans*. We find that, as in many meiotic programs, *C. elegans* spermatogenesis includes a chromosome aggregation or “karyosome” phase. This extended stage provides a period for chromosome and microtubule remodeling prior to the meiotic divisions. Our analysis identifies several gamete-specific features of the meiotic program that may contribute to the differential timing, pace, and mechanics of meiotic progression. Our findings provide a foundation for understanding how differentiation influences meiosis, which is an essential step in identifying universal features required for reproductive success in all organisms.

## Introduction

During either sperm or oocyte production, meiotic chromosomes undergo a continuum of similar events that are tightly regulated by the cell cycle. Meiosis starts with an extended G2 phase called meiotic prophase in which chromosomes first shorten (leptotene), then pair and assemble synaptonemal complexes (SC) (zygotene) before completing recombination (pachytene). Chromosomes then disassemble their SC (diplotene) and fully condense their bivalents (diakinesis). A subsequent transition from G2 to M is mediated by cell cycle kinases, including POLO and cdk-cyclin B, which drive nuclear envelope breakdown (NEBD), meiotic spindle assembly, and chromosome remodeling. Lastly, during M phase, two rounds of chromosome segregation generate haploid gametes with homologs segregating during the first ‘reductive division’ and sister chromatids segregating during the second. Since kinetochores of sister chromatids must orient towards the same spindle pole during the reductive division, some level of cohesion must be maintained between sister chromatids. Ultimately, proper meiotic segregation necessitates the combined activities of several regulatory proteins, including the Aurora B kinase [1],[2].

Concurrently, each sex executes the distinct developmental programs of spermatogenesis or oogenesis. Although there is still much to learn, comparative studies have documented several differences between meiosis of spermatogenesis and oogenesis. During meiotic prophase, germ cells interact with distinct gonadal environments [3]–[5] and are differentially regulated by apoptosis and cell cycle checkpoints [6]–[9]. For example, spermatocytes and oocytes vary in requiring an external signal to trigger the G2 to M transition, and many meiotic programs include a diapause at the end of meiotic prophase during which chromosomes aggregate to form a single, transcriptionally down-regulated mass called a karyosome [10],[11]. Later, during meiotic divisions, spermatocyte chromosomes segregate on centrally positioned centriole-based spindles to form four equally sized haploid spermatids [12] while oocyte chromosomes segregate on tiny, asymmetrically-positioned, acentriolar spindles to generate a single haploid oocyte and 2–3 degenerate polar bodies [8]. Challenges specific to spermatogenesis include the segregation of unpaired and/or heteromorphic sex chromosomes [13],[14] and the hypercompaction of the haploid sperm chromatin by systematic replacement of somatic histones with both histone variants and diverse protamine and protamine-like proteins [15],[16].

Several features make *Caenorhabditis elegans* ideal for analyzing sex-specific differences in meiosis. Many key proteins required for meiosis are evolutionarily conserved from worms to mammals [17]–[19]. Cells progressing through meiosis can be followed in a linear array along the length of the tube-like gonad in either isolated gonads or through the transparent body wall [20]. In hermaphrodites, a common pool of germ cells can generate either sperm or oocytes [21]. Studies of *C. elegans* oogenesis have provided insights regarding homolog pairing, meiotic recombination, desynapsis, and preparing gametes for meiotic divisions; in addition, they have identified key molecular markers for each meiotic stage [22]–[28]. Studies of *C. elegans* spermatogenesis have demonstrated its many assets as a model system, including a simplified differentiation program that occurs in the absence of accessory somatic cells or an extended post-meiotic differentiation period. Spermatogenesis-specific mutants can be studied in either males or hermaphrodites, which produce 200–300 sperm before switching to oocyte production [21],[29],[30]. However since few studies of *C. elegans* spermatogenesis have focused on meiotic prophase, molecular studies of this period will expand our understanding of fundamental events of meiosis and sex-specific modifications required in each sex.

The goal of this study was to explore how spermatogenesis-specific features coordinate with or modify the basic *C. elegans* meiotic program. In past studies, investigators have faced several challenges in linking underlying molecular events with cytological observations in late spermatogenesis. First, the rapid progression makes short-lived stages challenging to visualize in fixed preparations. Second, it is difficult to differentiate fine changes in the morphology of small meiotic chromosomes. To overcome these obstacles, we optimized preparation methods and identified molecular markers that differentiate specific stages of sperm meiosis. These markers define a broad set of cytological and molecular landmarks and enabled us to construct a detailed timeline of late meiotic prophase during *C. elegans* spermatogenesis. While this study identifies many aspects of meiosis that are common to both spermatogenesis and oogenesis, it also identifies multiple spermatogenesis-specific features. Our observations provide a foundation for understanding not only how cell-signaling pathways converge to control cell cycle progression and pace during meiosis but also how underlying molecular processes are differentially regulated between males and females.

## Results

### Chromatin morphology differs between spermatogenesis and oogenesis after pachynema

**Figure 1:**
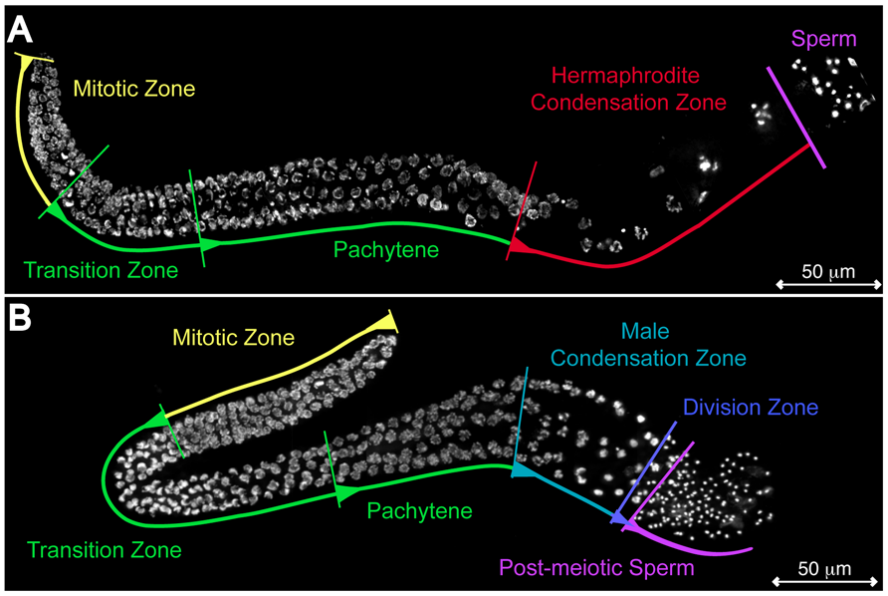
The progression of germ cell nuclei during gamete formation in *C. elegans*. DAPI-visualized nuclei in isolated and fixed gonads. (A) One arm of a bilaterally symmetric, two-armed, wild-type hermaphrodite gonad undergoing oogenesis and (B) a wild-type male gonad undergoing spermatogenesis. Regions of the gonad are labeled: mitotic (yellow), transition zone and pachytene (green), condensation zone of hermaphrodites (red) and males (teal), meiotic division zone (blue), and haploid spermatids (purple). The meiotic divisions of oocytes, which occur after fertilization, are not shown. Scale bars represent 50 µm.

In *C. elegans*, germ cells commit to oogenesis or spermatogenesis upon transition from mitosis to meiosis [31] but it was unknown when sex-specific differences in chromosome morphology could first be detected. To address this, we compared DAPI-stained nuclei in gonads isolated from adult males and hermaphrodites. Germ cells progress through early stages of meiosis while attached to a shared central core of cytoplasm known as the rachis [21]. Examination of nuclei undergoing DNA replication in the distal “mitotic region”, meiotic homolog alignment during leptotene/zygotene stages (crescent-shaped nuclei in the transition zones), or synapsis during the pachytene stage (basket-shaped nuclei) failed to reveal any obvious sex-specific differences in either nuclear size or shape (Figure 1A and 1B) [32].

Following pachytene, oocytes undergo a sequence of events that lead to their maturation [25],[27]. In late pachytene, many oocytes are culled by physiological germline apoptosis [33]. Surviving oocytes enlarge as they acquire large quantities of mRNA and protein from neighboring pachytene cells via cytoplasmic bridges [34] (Figure 1A). These oocytes become positioned to one-side and proceed single-file through the proximal gonad. At the same time, the chromosomes further condense to form compact bivalents. Thus, we refer to the region that includes the diplotene and diakinesis stages during both oogenesis and spermatogenesis as the ‘condensation zone’ (Figure 1). During this period, oocytes detach from the rachis [35] then breakdown their nucleolus [25],[28],[36]. They also down-regulate global transcriptional activity, as suggested by a dramatic decrease of serine 2 phosphorylation on the heptad repeat of the C-terminal domain of the large subunit of RNA polymerase II (pCTD-ser2), [37],[38]. As the nuclear envelope expands, oocyte chromosomes detach from the nuclear envelope as separate entities [21]. In response to a sperm-derived signal, the oocyte closest to the spermatheca, referred to as the -1 oocyte, undergoes NEBD then assembles its meiotic spindle [25],[26],[39].

**Figure 2:**
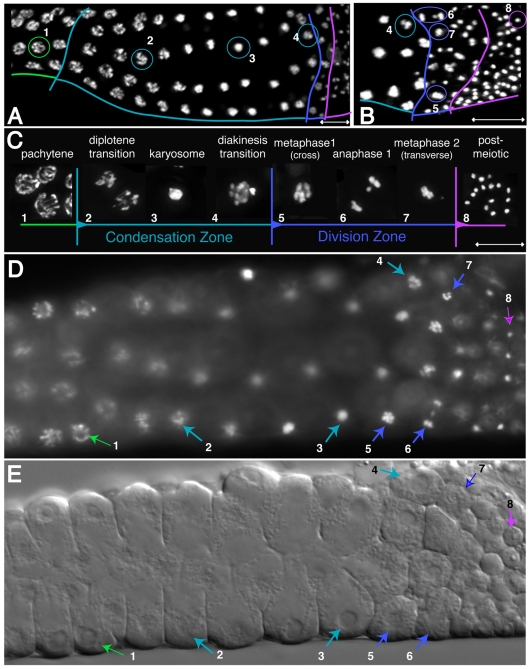
Karyosome formation during late spermatogenesis in *C. elegans*. Numbered nuclei highlighted by circles in (A) and (B) or arrows in (D) and (E) correspond to meiotic stages shown in (C). Pachytene (green), the condensation zone (teal), meiotic divisions (blue), post-meiotic region (purple). (A,B) DAPI-visualized nuclei in isolated and fixed male gonads (C) Enlarged images of DAPI-visualized nuclei in dissected and fixed male gonads. (D,E) A dissected and flattened non-fixed male gonad stained with the DNA dye Hoechst 33258 (D) and visualized by DIC optics (E). Scale bars represent 10 µm.

In contrast, post-pachytene spermatocytes in the condensation zone undergo a distinct series of morphological changes. First, the lack of physiological germline apoptosis [33] and dramatic cell growth enables spermatocytes to proceed in several single file rows around the rachis (Figure 1B and Figure 2A). During early chromosome condensation, chromosomes fail to separate into distinct entities. Instead they aggregate into a single mass within which individual chromosomes are not discernable, although DNA staining by DAPI often appears non-uniform (Figure 2A and 2C). Spermatocytes with this aggregated chromosome morphology are the most prevalent cells within the condensation zone (12–24/gonad) suggesting that spermatocytes exist in this state for an extended period (Figure 2A).

### Following diplotene, spermatocyte meiosis includes an extended karyosome stage

**Figure 3:**
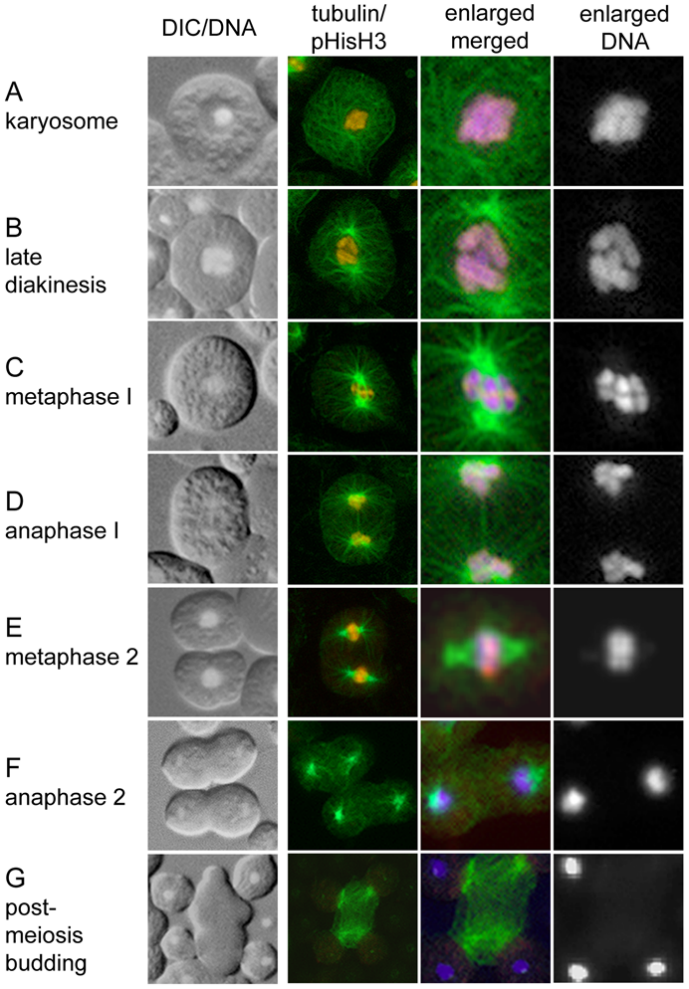
Changes in cell and microtubule morphologies distinguish spermatocytes that are entering and proceeding through the meiotic divisions. Left column (DIC/DNA) are unfixed and Hoechst-stained spermatocytes from flattened male gonad preparations viewed simultaneously under DIC and UV epifluorescence. Right columns are immunofluorescence analysis of methanol fixed spermatocytes doubled labeled with antibodies to α-tubulin (green) and pHisH3-ser10 (red) and stained with DAPI (blue in 2× enlarged merged image, white in enlarged DNA image). (A) karyosome, (B) late diakinesis, (C) metaphase I, (D) anaphase I, (E) metaphase II, (F) anaphase II, (G) budding figure. Primary spermatocytes are 12 microns in diameter.

**Figure 4:**
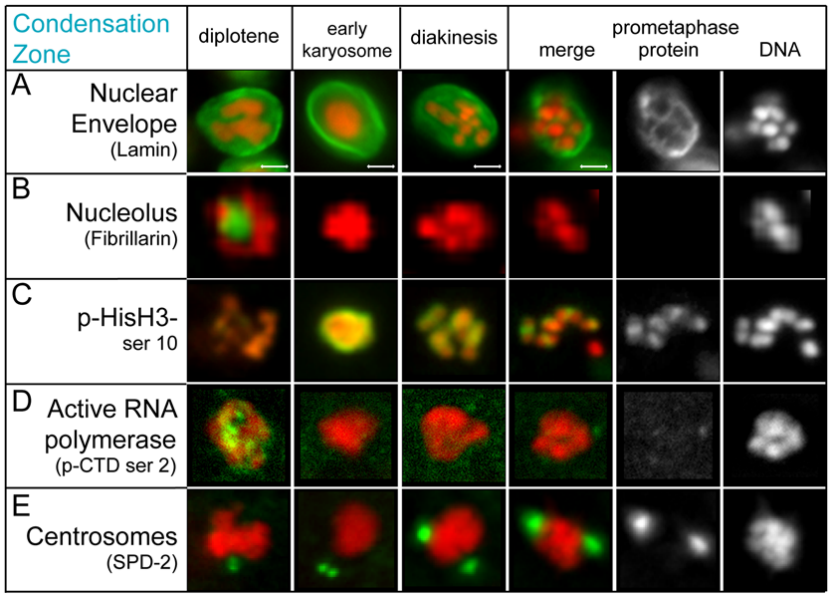
Immunolocalization of subcellular marker proteins defines events in the male condensation zone. Marker proteins, listed in parenthesis below, are shown in green. DNA is shown in red. (A) The nuclear envelope (lamin) disassembles during prometaphase (B) The nucleolus (fibrillarin) disappears before karyosome formation. (C) pHisH3-ser10 initiates in late diplotene, increasing in karyosomes, and shifts between homologous chromosomes during prometaphase. A spermatid chromatin mass is present in the bottom right of the prometaphase image. (D) High levels of active RNA polymerase levels (phosphorylated on the C-terminal domain on serine 2) decrease dramatically upon karyosome formation. (E) Centrosomes (SPD-2) separate and enlarge during the transition to diakinesis. Scale bars represent 2 µm. Panels in each row are sized the same as in (A).

To distinguish where this aggregated chromosome stage fits within the meiotic program in *C. elegans*, we correlated its occurrence with cytologically observable events before and after its formation. First, we used both differential interference contrast (DIC) optics and epifluorescence to examine non-fixed, flattened male gonads stained with the DNA dye Hoechst 33258 (Figure 2D and 2E). All spermatocytes in the condensation zone were attached to the rachis, while those in the adjacent division zone were detached. Proximal rachis-attached spermatocytes contained aggregated chromosomes within intact nuclear envelopes (Figure 2D and 2E and Figure 3A). Immunolocalization using antibodies against nuclear envelope markers, including lamin and nuclear pore complex proteins (Figure 4A and data not shown) confirmed this observation. Notably, nuclear envelope volume and shape remained relatively constant throughout the condensation zone, suggesting that nuclear envelope reduction is not driving chromosome aggregation. In parallel immunolocalization experiments using anti-α-tubulin antibodies, extensive microtubule networks throughout the cytoplasm also distinguished rachis-attached (Figure 3A) from rachis-detached spermatocytes transitioning to M-phase, which possessed prominent microtubule asters (Figure 3B).

Similar meiotic structures called “karyosomes” or “karyospheres”, in which paired homologs aggregate during or after diplotene, have been described during sperm and oocyte formation in organisms ranging from *Drosophila* to humans [10],[40],[41]. While karyosome function remains unclear, karyosome formation is most commonly associated with oogenesis in other organisms and is hypothesized to facilitate pre-division chromosome remodeling and grouping prior to meiotic divisions [10],[40]. Because both the morphology and timing of chromosome aggregation in *C. elegans* spermatogenesis correlates with karyosome formation in other organisms, we heretofore refer to this stage as the karyosome stage (Figure 2, Figure 3, and Figure 4).

After karyosome formation, spermatocytes detach from the proximal end of the rachis and rapidly enter meiotic divisions. In most gonads, we detected 0–2 nuclei transitioning from the karyosome stage to metaphase in two distinct stages. We define spermatocytes in late diakinesis as those that have newly detached from the rachis but possess intact nuclear envelopes and a slight degree of separation between their individual bivalents (Figure 2 and Figure 4A). Relative to chromosomes within oocytes at diakinesis (late condensation zone in Figure 1A), chromosomes within these spermatocytes at late diakinesis remained tightly confined within the smaller nucleus (Figure 2A–2C). We define prometaphase spermatocytes as those that have undergone partial or complete NEBD but whose chromosomes have not fully congressed to the metaphase plate (Figure 3B). We also routinely observed one or more metaphase I spermatocytes in each gonad (Figure 2 and Figure 3C).

### *C. elegans* karyosomes are transcriptionally down-regulated

To better understand karyosomes, we characterized the molecular events leading up to their formation. Prior to karyosome formation *C. elegans* sperm nuclear basic proteins (SNBPs) are incorporated into late pachytene chromosomes ([17] and data not shown). Immunolocalization studies using anti-fibrillarin (FIB-1) antibody revealed nucleoli abruptly disappearing before karyosome formation in late diplotene (Figure 4B). As in oocytes [28], nucleolar breakdown in spermatocytes coincided with the initiation of histone H3 (ser10) phosphorylation (pHisH3-ser10) (Figure 4C), a histone modification associated with pre-M-phase chromosome condensation [42]. pHisH3-ser10 levels were low in late diplotene but increased rapidly such that karyosome nuclei were the brightest staining within the gonad. Together, the incorporation of SNBPs, nucleolar breakdown and elevated pHisH3-ser10 levels suggest karyosome spermatocytes are largely transcriptionally down-regulated. Consistent with this interpretation, high levels of pCTD-ser2 in pachytene and diplotene nuclei abruptly decreased as karyosomes formed (Figure 4D) [38]. Karyosome formation therefore correlates with a decrease in global transcriptional activity.

### Disassembly of the synaptonemal complex exhibits sex-specific differences

To understand how karyosome formation fits in with events of late meiotic prophase, we examined karyosome formation relative to synaptonemal complex (SC) disassembly. The SC, a proteinaeous scaffold, assembles prior to pachynema to facilitate and regulate recombination [18],[43]. SCs are composed of two structures referred to as axial/lateral elements and central elements. Lateral/axial elements are composed of proteins, such as HIM-3, that polymerize between sister chromatids along each homologous chromosome length [44]. Central region proteins, like SYP-1, link the axes between homologous chromosomes [45], and the loss of SYP-1 marks desynapsis.

**Figure 5:**
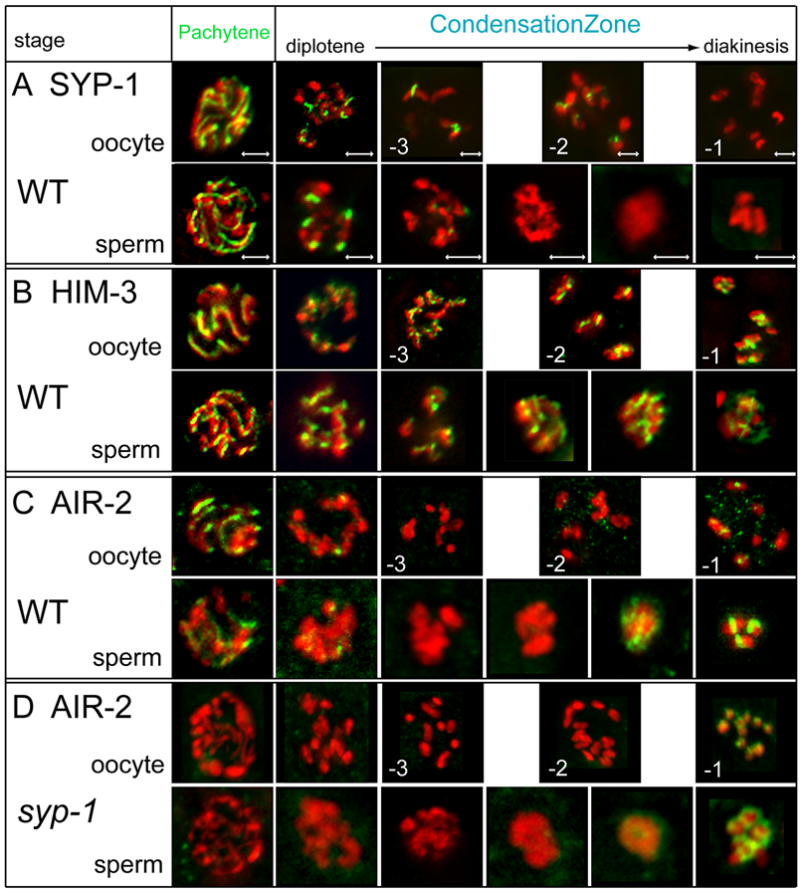
Synaptonemal complex (SC) protein disassembly in sperm and oocyte meiosis. Immunolocalization of SC proteins is shown in green and DNA in red. (A) The SC central element protein, SYP-1, departs asymmetrically from chromosomes early in the transition from diplotene to diakinesis during spermatogenesis but retained on oocyte chromosomes until late diakinesis. (B) The axial/lateral element protein HIM-3 associates to chromosomes similarly in sperm and oocyte condensation zones. (C) AIR-2 is present along each chromosomal axis in pachytene and early diplotene nuclei in oogenesis and spermatogenesis. In oogenesis, AIR-2 is not detectable in late diplotene or early diakinesis but reappears in late diakinesis on the short arms of each bivalent. During spermatogenesis, AIR-2 localizes to discrete regions on karyosome then shifts in localization to the short arms of the bivalents during diakinesis. (D) AIR-2 is absent during pachytene in *syp-1(me17)* mutants in both oogenesis and spermatogenesis. AIR-2 reappears during spermatogenesis, but is mislocalized in an uneven distribution around univalents. AIR-2 during oogenesis reappears during diakinesis only in *syp-1(me17)* mutants mated with WT males in the -1 oocyte (unmated *syp-1(me17)* -1 oocyte is not shown) and is also mislocalized. Scale bars represent 2 µm. Panels in rows corresponding to sperm or oocyte are sized the same as in (A).

Interestingly, oocytes and spermatocytes differ in the dynamics of central element disassembly. In post-pachytene oocytes, SYP-1 becomes progressively restricted to axes distal to the chiasmata with regions of SYP-1 retained in all but the -1 oocyte. Complete SYP-1 removal occurs only after nucleolar breakdown and the appearance of pHisH3-ser10 [24],[25],[42] (Figure 5A oocyte). In post-pachytene spermatocytes, SYP-1 undergoes a phased departure through diplotene but disappears prior to karyosome formation, well before chromosomes are fully condensed (Figure 5A sperm). Notably, SYP-1 removal in late diplotene spermatocytes occurs just before nucleolar breakdown and the appearance of pHisH3-ser10 staining (Figure 4B and 4C, Figure 5A, and data not shown). Thus SYP-1 is lost earlier in spermatocytes than it is in oocytes relative to meiotic stage, nucleolar breakdown, and degree of chromosome compaction. Such sex-specific differences in the timing of desynapsis relative to other late meiotic events may reflect fundamental differences in sperm chromatin composition and structure and hint at additional underlying sex-specific alterations in the corresponding meiotic machinery.

The observation that karyosome formation initiates directly following SC disassembly suggested a possible link between the two events. To test whether SC formation was a prerequisite for karyosome formation, we analyzed karyosome formation in males with mutations in core SC elements. Despite their SC defects [45],[46], both *syp-1(me17)* and *him-3(gk149)* males produced spermatocytes that assembled karyosomes (Figure 5D and data not shown). Thus, the presence of core SC components, and presumably the assembly of the SC, is not a prerequisite for spermatocyte karyosome formation.

### Chromosomes in karyosomes retain structural organization

In contrast to SYP-1, the lateral element component, HIM-3, remains associated with oocyte chromosomes during the coiling-based process of diplotene chromosome shortening (Figure 5B oocyte) [24],[44],[45]. As such, we studied HIM-3 localization in spermatocytes as a marker for chromosome organization. HIM-3 assembled along the lengths of pachytene chromosomes and remained associated throughout the condensation zone. HIM-3 decorated the karyosome in distinct stripes then shifted to an X-shaped pattern along chromosome axes as individual bivalents resolved, similar to the localization pattern observed on chromosomes of oocytes at diakinesis (Figure 5B sperm). This pattern suggests that homologs remain aligned during and following karyosome formation. Although HIM-3 was previously reported to persist at high levels on metaphase I spermatocyte chromosomes [44], we detected a dramatic metaphase reduction of HIM-3 levels. We suspect that previous investigators, using only DNA or immunostaining of SC components, were unable to distinguish karyosome from metaphase I spermatocytes. In this adjusted analysis, HIM-3 localization patterns are similar during oogenesis and spermatogenesis.

### Changes in AIR-2 localization reveal that the karyosome is highly dynamic

Another key player in meiotic progression is the aurora-like kinase AIR-2 [47]. As one of several “chromosomal passenger proteins”, AIR-2 mediates meiotic and mitotic chromosome condensation, chromosome-kinetochore attachments, sister chromatid release, and cytokinesis. During oogenesis, AIR-2 colocalizes with SYP-1 along the axes of pachytene chromosomes and then departs during early diplotene (Figure 5C oocyte) [24]. AIR-2 reassociates to the short arms of the bivalents in the -1 oocyte in the same pattern as the recently departed SYP-1 only when a signal from sperm residing in the spermatheca triggers the G2 to M transition (Figure 5C oocyte) [1],[24],[28],[48],[49]. Without sperm, hermaphrodites accumulate oocytes at diakinesis that are AIR-2 negative [49].

During spermatogenesis, AIR-2 localized along the axes of pachytene chromosomes and departed during early diplotene as in oogenesis (Figure 5C sperm). However, AIR-2 exhibited a phased reassociation to chromosomes during karysome formation reaching high levels on late karyosome nuclei and concentrating in discrete regions visible on the external surface. As spermatocytes detached from the rachis and transitioned to prometaphase, AIR-2 localized on the short arm of the bivalents [2],[48]. This shift in AIR-2 localization may reflect active cycles of AIR-2 unbinding and rebinding the chromatin or, alternatively, the passive movement of chromatin-bound AIR-2 on structurally dynamic chromosomes. Overall, we found AIR-2 reassociation in spermatogenesis is distinct from oogenesis – it is highly dynamic, signal independent, and occurs earlier.

The apparent “exchange” of SYP-1 for AIR-2 within oocytes at late diakinesis suggested SYP-1 guides AIR-2 localization [18],[24]. However, the temporal gap between SYP-1 loss and AIR-2 rebinding during spermatogenesis seemed inconsistent with this model. To test the dependency of AIR-2 chromosome association on SYP-1, we examined AIR-2 localization in *syp-1(me17)* homozygous mutant males and hermaphrodites. In *syp-1* spermatocytes, AIR-2 was undetectable on pachytene chromosomes, yet AIR-2 still reassociated with karyosomes (Figure 5D). However instead of concentrating in discrete regions, AIR-2 bound diffusely thoughout the *syp-1* karyosome mass, before binding unevenly to chromosomes at diakinesis.

In *syp-1(me17)* hermaphrodites, AIR-2 was undetectable in oocytes at the pachytene and diakinesis stages [24] (Figure 5D oocyte and data not shown). To distinguish whether the lack of AIR-2 staining in -1 oocytes in *syp-1* mutants reflected the lack of “guiding” SYP-1 or the lack of a sperm signal from defective *syp-1* sperm, we mated *syp-1(me17)* mutant hermaphrodites to wild-type males. In the presence of normal sperm, the *syp-1* oocytes were triggered to mature and AIR-2 was detectable on -1 oocyte chromosomes, albeit in an abnormal pattern (Figure 5D oocyte). These results suggest that SYP-1 is required in a sex-independent manner to recruit AIR-2 to pachytene chromosomes. Later, SYP-1 is dispensable for recruiting AIR-2 to chromosomes but required for properly localizing AIR-2 to the short arms of bivalents at diakinesis.

Our analysis also refines the role of AIR-2 in phosphorylating serine 10 of histone H3 during the transition to meiotic divisions. Previous work has shown AIR-2 is required for HisH3-ser10 phosphorylation in both maturing *C. elegans* oocytes [42] and mouse spermatocytes [50]. However, in *C. elegans* spermatocytes, pHisH3-ser10 not only appeared earlier than AIR-2 but the two markers also exhibited distinct localization patterns on chromosomes during the diplotene, karyosome, and diakinesis stages. This suggests that another kinase is responsible for HisH3-ser10 phosphorylation in late diplotene and karyosome spermatocytes (Figure 5C, Figure 3B and 3C, and Figure 4C). When the two proteins co-localize during prometaphase, AIR-2 may assume the role of phosphorylating HisH3-ser10 (Figure 4C).

### The transition to M-phase initiates in late karyosomes

The G2 to M transition marks the end of diakinesis and an irreversible commitment to meiotic divisions [25],[27],[28]. Typical transitional events include NEBD, changes in microtubule dynamics, centrosome separation, and several pre-division chromosome modifications. In *C. elegans* oocytes, the G2 to M transition initiates with nucleolar breakdown and HisH3-ser10 phosphorylation in the -3 and -2 oocytes followed by AIR-2 recruitment and NEBD in the -1 oocyte [28]. Oocytes of *C. elegans* and most other organisms lack centrioles [51]–[53], thus their meiotic G2 to M transition does not involve centrosome nucleation and separation, and chromosome-mediated spindle assembly initiates only after NEBD.

**Figure 6:**
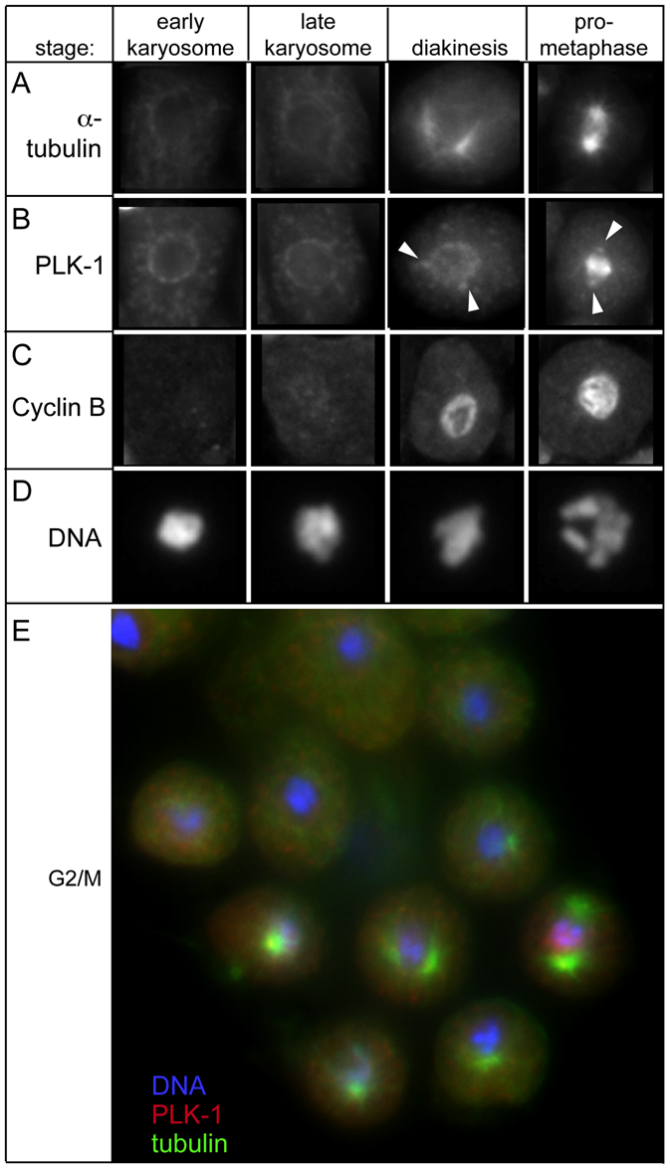
Activation of signaling pathway components during the G2/M transition in spermatogenesis. (A) α-tubulin costaining with (B) PLK-1. PLK-1 shifts from a ring around the nuclear envelope in diplotene and karyosome nuclei and then localizes to centrosomes (arrowheads) during the transition to diakinesis. In prometaphase, PLK-1 is detected both on centrosomes and on chromosomes. (C) Cyclin B levels increase during diakinesis and stays localized around chromosomes in prometaphase. (D) 2× enlarged images of DNA from cyclin B immunostained imaged in (C). (E) Sequence of karyosome to budding figure spermatocytes from flattened and fixed male gonad preparations immunostained with α-tubulin (green) and PLK-1 (red). DNA is shown in blue.

Because spermatocytes have centrioles, we anticipated that microtubule reorganization would mark the G2 to M transition. In all diplotene and most karyosome spermatocytes, immunostaining for SPD-2, a core component of both active and inactive centrosomes [52],[54], revealed pairs of tiny, side-by-side SPD-2 foci (quiescent centrosomes) situated on one side of the nucleus (Figure 4E) at a stage when the spermatocyte cytoplasm was filled with unfocused microtubules (Figure 3A and data not shown). In late karyosome spermatocytes, SPD-2 foci enlarged, indicating centrosome activation, and initiated separation. Cortical microtubule superstructures and small pairs of microtubule asters ranging from 0–90° of separation were also visible (data not shown). In rachis-detached spermatocytes, microtubule asters continued to enlarge and separate until, by NEBD, they were fully opposed and the only remaining microtubule superstructure (Figure 3B and 3C, Figure 6A). Thus, the G2 to M transition of spermatogenesis is associated with nucleation and separation of microtubule asters, exit from the karyosome stage, and rachis detachment. In contrast to oogenesis, spermatocyte meiotic spindle assembly is largely completed prior to NEBD.

Having discovered that microtubule aster assembly and separation initiates in late karyosome spermatocytes, we also investigated the distribution of the cell cycle regulator polo-like kinase (PLK-1) [55], which has been implicated in cell division processes including mitotic spindle formation and mitotic entry [56]. Interestingly, repression of PLK by the PLK binding protein Matrimony maintains the G2 karyosome state in *Drosophila* oocytes [57]. In *C. elegans* diplotene and early karyosome spermatocytes, PLK-1 concentrates in a ring around the nuclear envelope and punctate structures throughout the cell (Figure 6B and 6D). In late karyosome spermatocytes, PLK-1 localized to active centrosomes. After NEBD, PLK-1 continued to associate with centrosomes but also bound to metaphase chromosomes. This dynamic pattern is consistent with PLK-1 mediating microtubule nucleation [58] and centrosome separation [59]. It also suggests that PLK-1 relocalization marks the G2 to M transition in spermatocytes. Consistent with PLK-1 either activating or being activated by the universal regulator of the G2 to M cell cycle transition, Cdk1-cyclinB, cytoplasmic levels of cyclin B increase throughout the karyosome stage, and cyclin B switches to a predominantly nuclear distribution near the time of rachis detachment (Figure 6C and 6D).

### Assembly of kinetochore components show sex-specific differences

Because spermatocytes and oocytes differ in chromatin composition and meiotic spindle structure and assembly, we anticipated that kinetochores, which link chromosomes to microtubules, might also vary in structure or assembly. During *C. elegans* mitosis, kinetochores are large, plaque-like structures, reflecting the holocentric nature of their chromosomes [60],[61]. Studies of mitotic cells suggest a stepwise assembly of kinetochores [62],[63] in which the evolutionarily conserved inner kinetochore components HCP-3^CENP-A^ and HCP-4^CENP-C^ establish a specialized chromatin base for the association of outer kinetochore proteins, which interface with spindle microtubules. However, meiotic-specific kinetochore structures may be required for orienting sister chromatids towards microtubules from the same spindle pole for the first meiotic division. Kinetochores of spermatocytes and oocytes may also differ since spermatocyte spindles are centriole-based while oocyte spindles are not. In fact, proteomic studies have identified gamete-specific differences in the levels of *C. elegans* kinetochore proteins [17]. Specifically, HCP-4^CENP-C^ was enriched in spermatogenic chromatin while HCP-3^CENP-A^ was enriched in oogenic chromatin. Similarly, the outer kinetochore protein HCP-1 was detected in chromatin preparations from oogenic germ cells but not from sperm.

**Figure 7:**
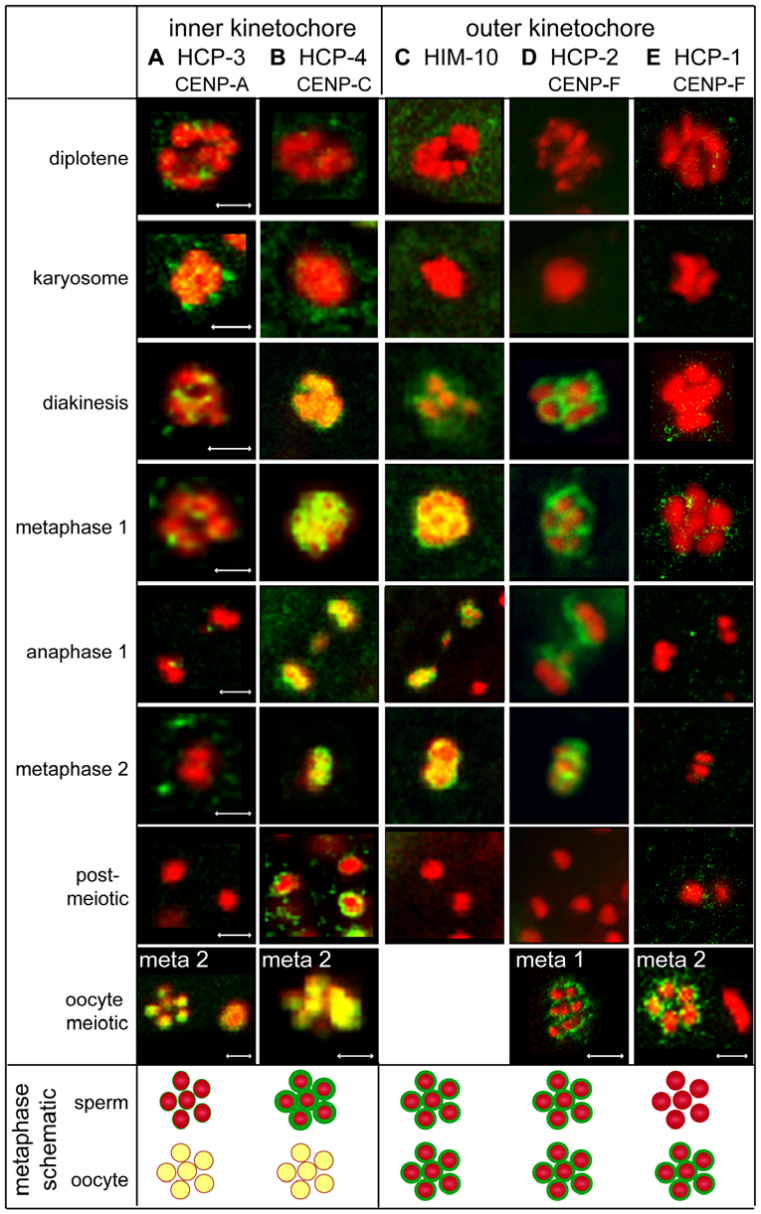
Inner and outer kinetochore components are differentially enriched and localized in sperm meiosis compared with oocyte meiosis. Immunolocalization of kinetochore components (green) and DAPI-stained DNA (red) during spermatogenesis and in metaphase oocytes. Yellow coloration in images and the schematics indicates extensive co-localization. (A) Barely detectable levels of the inner kinetochore component HCP-3^CENP-A^ encase spermatocyte chromosomes, but is at high levels distribute evenly all over oocyte chromosomes. (B) High levels of the inner kinetochore component HCP-4^CENP-C^ surround spermatocyte chromosomes starting in diakinesis and persist after meiosis is complete. HCP-4^CENP-C^ distributes evenly all over oocyte chromosomes. (C) The outer kinetochore components HIM-10 and (D) HCP-2^CENP-F^ surround spermatocyte and oocyte chromosomes at high levels. (E) The outer kinetochore component HCP-1^CENP-F^ is not detectable in sperm meiosis but surrounds chromosomes during oocyte meiosis. Scale bars represent 2 µm. Panels in each column are sized the same as (A) except as marked for oocyte meiotic chromosomes.

In this study, immunoanalysis of five different kinetochore proteins revealed striking sex-specific differences in the relative levels of specific kinetochore proteins. Although we found high levels of the inner kinetochore protein HCP-3^CENP-A^ and the outer kinetochore protein HCP-1 in oocytes, these proteins were barely detectable in spermatocytes (Figure 7A and 7E). Conversely, HCP-4^CENP-C^ was highly abundant in spermatocytes (Figure 7B). Because inner kinetochore components are intimately incorporated into chromatin, the near absence of detectable HCP-3^CENP-A^ on spermatocyte chromosomes suggests that spermatocytes and oocytes differ in the organization of their kinetochore components. The CENP-F homologs, HCP-1 and HCP-2 are thought to function non-redundantly in mitotic spindle checkpoint assembly but redundantly in mitotic chromosome segregation [64]–[66]. The near absence of detectable HCP-1 suggests that HCP-2 may function non-redundantly in spermatogenesis (Figure 7D and 7E). Notably, while all other kinetochore proteins were lost after anaphase II, HCP-4^CENP-C^ perdured, encircling the post-meiotic sperm chromatin mass.

Localization patterns revealed similarities and differences in the kinetochores of spermatocytes and oocytes. Interestingly, HCP-3^CENP-A^ or HCP-4^CENP-C^ localization differed strikingly in spermatocytes and oocytes (Figure 7A and 7B). In oocytes, these proteins co-localize with the chromatin in an “inner” pattern, while in spermatocytes they “surround” the spermatocyte chromosomes in a pattern previously described for outer kinetochore proteins. Antibody inaccessibility is unlikely to account for this difference as co-immunostaining with anti-histone H1 antibody showed an even distribution of histone H1 on meiotically dividing chromosomes (data not shown). HIM-10, an evolutionarily conserved outer kinetochore protein surrounded the spermatocyte and oocyte chromosomes in a similar manner (Figure 7C and [61]). The outer kinetochore protein HCP-2 also surrounded spermatocyte chromatin, yet in comparison to HIM-10, its minimal co-localization with the chromatin suggests a less intimate association (Figure 7C and 7D). HCP-2 localized completely between separating chromosomes in anaphase I then both asymmetrically and symmetrically on metaphase II chromosomes, indicating HCP-2 relocalizes from one side of the chromosomes to surround DNA prior to meiosis II (data not shown). Thus in spermatocytes, inner kinetochore proteins exhibit a localization patterns more similar to outer kinetochores.

This differential enrichment and localization of kinetochore components is the first evidence suggesting that the molecular machinery required for chromosome segregation in spermatocytes may differ from that in either oocytes or mitotically dividing cells. Sex-specific differences in the molecular composition of meiotic kinetochores may reflect differences either in the structure of the meiotic chromosomes or in the molecular requirements for interacting with structurally distinct meiotic spindle structures.

### Chromatin and microtubule dynamics accurately stage sperm meiotic divisions

The presence or absence of centrosomes not only affects the relative timing of meiotic spindle assembly but also influences the structure and mechanics of the spindles. In *C. elegans*, oocyte chromosomes apparently slide to metaphase congression between bundled microtubules as they segregate on a barrel-shaped, acentriolar spindle [67],[68]. Anaphase movements are also distinctive with short-distance movements from the midline to the poles followed by further separation as the zone of midbody microtubules lengthens between the chromosome plates [26],[68].

Although the meiotic cell divisions during *C. elegans* spermatogenesis have been described [30], we used improved immunocytological methods to stage small and scarce dividing spermatocytes. To do this we characterized chromosome morphology in combination with DIC cell morphology [30] or microtubule dynamics [69] (Figure 3 and Table 1) in flattened gonad preparations. Centriole based microtubule asters are first detectable in late karyosome spermatocytes and extend throughout the cell by the end of diakinesis as centrosomes separated to opposite sides of the nuclear envelope (Figure 3B). Upon NEBD chromosomes attached to microtubules and congressed to form a metaphase I plate in which the X-chromosome is surrounded by autosomes (Figure 3C). Metaphase I spermatocytes exhibited prominent astral microtubules. At anaphase I, spindle poles separated and disjoined homologs moved to opposite poles, with the X-chromosome frequently lagging between between (Figure 2C and Figure 3D). Cytokinesis following anaphase I was often incomplete with a small cytoplasmic bridge connecting secondary spermatocytes [30]. Transition from anaphase I to metaphase II occurred without intervening chromosome decondensation and with metaphase II spindle poles setting up directly adjacent to the metaphase II plates (Figure 3E). During anaphase II, microtubules concentrated at the spindle poles with chromosomes in between; midbody microtubules were not prominent (Figure 3F). After a transient, shallow cleavage furrow formed and rapidly regressed, spermatids budded from a central residual body that accumulates materials not needed by mature sperm, including the bulk of the microtubules (Figure 3G; [30]). During this final phase, the haploid chromosome complement quickly condensed further, forming a tiny, spherical, highly refractive chromatin mass. Importantly, these studies distinguish anaphase II from the asymmetric “budding division” that partitions spermatids from residual bodies as distinct and sequential events. Overall, the progression of meiotic division stages can now be reliably distinguished by a combination of chromatin, cell, and microtubule morphology (Table 1).

**Table 1:** Summary of chromatin and microtubule characteristics of meiotic divisions during spermatogenesis.

Meiotic Stage	Chromatin morphology	Microtubule Characteristics
Diplotene	Condensing bivalents associated with nuclear envelope	Network throughout cytoplasm
Karyosome	Chromosomes aggregated in a single mass within nuclear envelope	Network throughout cytoplasm. Also tiny asters in late karyosome spermatocytes
Diakinesis	Slight separation of individual chromosomes	Large asters in near or full opposition. Presence of nuclear envelope prevents microtubule-chromosome contact
Prometaphase	Further separation of chromosomes	Spindle microtubules extend to chromosomes after nuclear envelope breakdown
Metaphase I	Chromosomes aligned in pentagonal plate with X chromosome in center	Large spindle within large single cell. Microtubules attached to chromosomes
Anaphase I	Two flattened plates often with lagging X chromosome	Single anaphase spindle within large single cell. More astral than mid-body microtubules.
Metaphase II	Chromosomes aligned in a small pentagonal plate	Two smaller side-by-side or nearby metaphase spindles
Anaphase II	Two pairs of flattened plates.	Two smaller side-by-side or nearby anaphase spindles. Microtubules radiate in all directions from centrosome.
Budding Figure	Compact, round, haploid nuclei	Polymerized microtubules within a central residual body

### During the meiotic divisions, key kinases localize in a non sex-specific pattern

**Figure 8:**
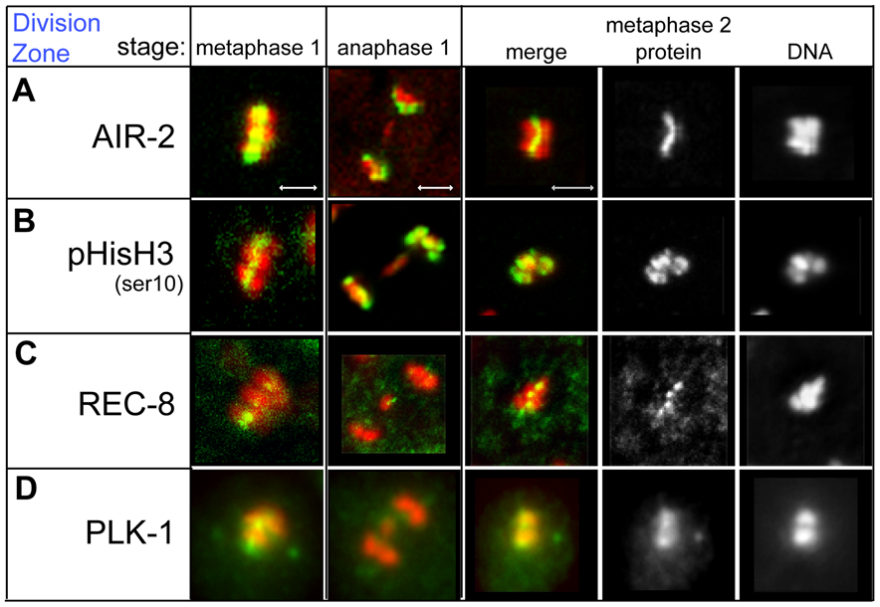
Immunolocalization of chromosome segregation markers during spermatocyte meiotic divisions. Proteins involved in chromosome segregation are green and DNA is red. (A) AIR-2 localizes to the short arms of bivalents during metaphase I and between sister chromatids during metaphase II. (B) p-HisH3-ser10 concentrates on regions bound by AIR-2. (C) The cohesin protein REC-8 is detectable along the equatorial plane on metaphase I and II plates. (D) PLK-1 protein is present on centrosomes throughout the meiotic divisions, chromosomes during metaphase, and between chromosomes during anaphase. Scale bars in (A) represent 2 µm. Panels in each column are sized the same as (A).

In parallel studies, we studied the localization patterns of factors that facilitate meiotic divisions. These include the kinases AIR-2 and PLK-1, as well as the AIR-2 targets pHisH3-ser10 and the meiotic cohesin protein REC-8. AIR-2 phosphorylates REC-8, which is present both between sister chromatids and between homologs, marking it for removal during the sequential metaphase-to-anaphase transitions [70]. Meiotic chromosome segregation thus requires AIR-2 to specifically localize between paired homologs during meiosis I and only relocalize between sister chromatids during meiosis II [2],[48],[71]. In fact, localization of AIR-2, pHisH3-ser10, and REC-8 to the mid-bivalent during spermatogenic meiotic divisions matches that described for meiotically dividing oocytes (Figure 8A–8C) [71]. PLK-1, which regulates both microtubule and cell cycle events during meiosis, first associated with late karyosome centrosomes and persisted there through anaphase II (Figure 6 and Figure 8D). PLK-1 also bound to chromatin during prometaphase and metaphase II but also localized between segregating chromosomes during anaphase I and II (Figure 8D). The dynamic localization of PLK-1 may enable it to promote cohesion release during the metaphase-to-anaphase transition [72] and subsequently promote cytokinesis during anaphase [73]. Taken together, the localization of AIR-2, pHisH3-ser10, REC-8, and PLK-1 in meiotically dividing spermatocytes can distinguish sub-stages of the meiotic divisions. Furthermore, while AIR-2 and PLK-1 exhibit sex-specific localization patterns before the meiotic divisions; their localization patterns during the meiotic divisions are remarkably non sex-specific.

## Discussion

**Figure 9:**
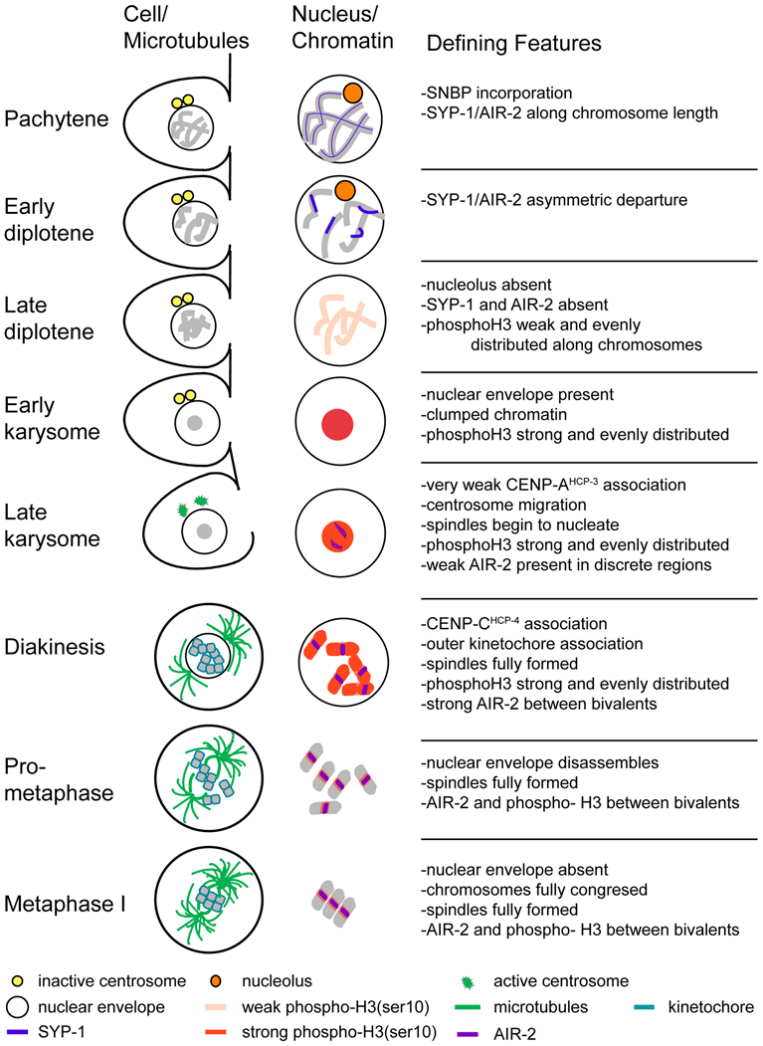
A summary of the progression of nuclear events during late sperm formation.

The timeline of late meiotic prophase during spermatogenesis in *C. elegans* provided here uniquely ties changes in chromosome morphology to germ cell cellularization, subnuclear structure disassembly, microtubule spindle assembly, and cell cycle transitions (Figure 9). We found that spermatocyte chromosomes undergo distinctive morphological changes after incorporating sperm-specific chromatin structural proteins. The most striking is karyosome formation, a staging ground where chromosomes aggregate, transcription is largely down-regulated, and multiple kinases are sequentially activated to prepare for meiotic divisions. This study also documents how spermatocytes differ from oocytes in the dynamics of desynapsis, the structure of their kinetochores, and the assembly and morphology of their meiotic spindles. These numerous sperm-specific features bring to light how the underlying molecular machinery required for meiosis is differentially regulated in each sex.

### Sperm-specific processes may facilitate accelerated meiotic progression

In *C. elegans*, sperm and oocyte meiosis occur at remarkably different rates. Meiotic prophase lasts 54–60 hours during oogenesis and only 20–24 hours during spermatogenesis [74]. How is meiotic progression accelerated during sperm formation? While previous studies suggest the lack of a DNA damage checkpoint during spermatogenesis shortens the pachytene period [74],[75], our studies reveal sperm-specific components and instances of overlapping developmental sub-programs that could potentially speed meiotic progression. For example, the sperm-specific presence of centrioles allows meiotic spindle assembly prior to NEBD [51],[76]. These preformed spindles could accelerate the initiation of chromosome segregation in spermatocytes compared to oocytes, which must default to an alternate, chromosome directed mode of spindle assembly that can only begin after NEBD [77],[78].

A second key event in spermatogenesis is the shaping and compaction of spermatid chromatin, which typically occurs during an extended period of post-meiotic differentiation [79],[80]. *C. elegans* spermatids lack a prolonged post-meiotic differentiation phase [81], yet achieve similar chromatin compaction. Shifting key events earlier may facilitate this process. For example, while mammals incorporate variant histones during meiosis and protamines after meiosis, *C. elegans* incorporates all currently known SNBPs at the end of pachytene while chromatin structure remains relatively more accessible [17]. Additionally, once sperm chromatin is condensed for meiotic divisions, it does not decondense for a post-meiotic round of transcription. Instead, final compaction of the haploid chromatin is reduced to a quick step following anaphase II, suggesting that required components may be pre-loaded and merely require an as yet unknown post-translational switch.

Down-regulation of transcription is also shifted to an earlier stage in *C. elegans* sperm formation. During *C. elegans* oogenesis, nucleolar breakdown, HisH3-ser10 phosphorylation, and AIR-2 reassociation are coupled to the G2 to M transition [25],[28],[49] and delayed when cell cycle progression is halted by either the absence of sperm or depletion of *cdk-1* [28]. In contrast, during spermatogenesis these events occur during the earlier diplotene to karyosome transition with AIR-2 reassociation following. Thus, events associated with transcriptional down-regulation appear to be uncoupled from the G2 to M transition in spermatocytes. Consistent with this model, spermatocytes still form compact, pHisH3-ser10 positive, chromatin masses even when the G2 to M events of spindle formation and NEBD are blocked by dominant “always on” mutations in the cell cycle regulator *wee-1.3* [82]. Our studies predict that these mutant spermatocytes have completed the diplotene to karyosome transition but are subsequently arresting at the karyosome stage.

### Discovery and analysis of a karyosome stage in *C. elegans* spermatogenesis

We have found *C. elegans* spermatocytes form karyosomes, a feature of meiosis in more than 120 species including *Drosophila*, mouse and humans [10],[41],[83],[84]. While karyosomes are proposed to prepare and gather chromosomes prior to meiotic divisions, our studies indicate that these functions can also be important for chromosomes that are holocentric and/or segregate on centriole-based spindles. Our studies also suggest that karyosome chromatin is both highly structured and dynamic; karyosome chromosomes exhibit organized stripes of the SC axial/lateral element protein HIM-3 and the aurora kinase, AIR-2. Disruption of these patterns in the absence of proper SC formation suggests the chromosomal superstructure of karyosomes may “lock in” SC-related organizational information that would otherwise be lost after desynapsis.

As in other organisms, *C. elegans* karyosomes form during or after diplonema [10]. We further found that karyosome formation coincides with nuclear envelope detachment, SC central element protein (SYP-1) loss, HisH3-ser10 phosphorylation, and transcriptional down-regulation. *Drosophila* oocytes undergo a similar suite of events during karyosome formation and these events are collectively disrupted by mutations in the nucleosome histone kinase NHK-1, also known as vaccinia related kinase VRK-1 [83]. Known substrates of NHK-1/VRK-1 include histone H3-ser10 [85], as well as histone H2A-thr119 [83] and the chromatin-nuclear envelope linker BAF-1 [86]. Thus, NHK-1/VRK-1 is a prime candidate for linking karyosome formation to other cellular events in *C. elegans* spermatogenesis. Unfortunately, germline proliferation defects in *nhk-1/vrk-1* mutants have thus far precluded us from testing this prediction (data not shown).

Other proteins, like the cell cycle regulator *wee-1.3*, may control whether late prophase chromosomes aggregate into karyosomes or disperse, as in *C. elegans* oocytes at diakinesis [67]. In developing oocytes, RNAi depletion of *wee-1.3* causes precocious maturation involving premature nucleolar breakdown and histone H3 phosphorylation [25],[28]. Strikingly, these mutant oocytes exhibit chromosome aggregation reminiscent of karyosome formation, as well as ectopic microtubule aster formation prior to NEBD [28]. Thus *wee-1.3* is an excellent candidate for an oocyte-specific regulator that delays the G2-to-M transition until oocytes are properly prepared for meiotic divisions and fertilization.

Spermatocytes and oocytes also differ in rachis detachment timing. While rachis detachment accompanies the diplotene to diakinesis transition of oogenesis [35], it accompanies the G2 to M transition of spermatogenesis (this paper). Transcriptionally repressed, detached spermatocytes lack somatic support while rachis-detached oocytes at diakinesis endocytose yolk proteins from the pseudocoelom and maintain gap junction contact with surrounding somatic sheath cells until ovulation [87]. Thus, for spermatocytes, rachis detachment may represent a critical point of “cellular independence”. For oocytes, the analogous point is not rachis detachment but ovulation.

### Spermatocyte kinetochores may reflect underlying differences in chromatin structure

Though late prophase spermatocytes and oocytes exhibit many differences, their metaphase I chromosomes have remarkably similar patterns of AIR-2, pHisH3-ser10, REC-8, and PLK-1 localization. Thus an open question was whether kinetochores also differ in a gamete specific manner. Our finding that spermatocyte and oocyte kinetochores do differ in molecular composition and localization patterns suggests kinetochore structure may adapt to reflect sex-specific differences of meiotic spindles and underlying chromatin structure.

Meiotic kinetochores also apparently differ from their mitotic counterparts. On mitotically dividing chromosomes, the inner kinetochore protein HCP-3^CENP-A^ is required to recruit HCP-4^CENP-C^ [62],[63]. On meiotically dividing oocyte chromosomes, HCP-3^CENP-A^ and HCP-4^CENP-C^ are present at high levels, but their role is controversial. When RNAi was used to deplete HCP-3^CENP-A^ and HCP-4^CENP-C^, live studies of chromosome segregation in GFP-histone tagged oocytes suggested that HCP-3^CENP-A^ and HCP-4^CENP-C^ were dispensible for oocyte meiosis [88]. However, analyses using fluorescence *in situ* hybridization (FISH) or restriction fragment length polymorphisms (RFLPs) to tag individual chromosomes revealed reproducible segregation defects (A. Severson and B. Meyer, *pers. comm.)*. Our own studies add to the puzzle, since HCP-3^CENP-A^ was barely detectable on spermatocyte chromosomes while HCP-4^CENP-C^ was highly enriched. This may reveal that very low levels of HCP-3^CENP-A^ may be sufficient to recruit HCP-4^CENP-C^. Alternatively, histone replacement and SNBP incorporation during late pachynema [17] may alter chromatin structure and consequently influence subsequent chromatin-based events, like kinetochore assembly. For instance, incorporation of the histone variant H2A.X proved essential for heterochromatic chromatin formation of the XY body [89]. Likewise, protamine or protamine-like proteins package DNA in a non-nucleosomal configuration [16],[90]. Therefore, SNBP-based chromatin packaging may itself provide a sufficient platform for HCP-4^CENP-C^ recruitment or maintenance. Indeed, following meiotic divisions and the departure of all other kinetochore proteins, HCP-4^CENP-C^ remains bound to sperm chromosomes.

### Landmarks of *C. elegans* spermatogenesis as a framework for comparative studies

This study describing the dynamics of key markers throughout spermatogenesis establishes guidelines for staging *C. elegans* spermatocytes and characterizing spermatogenesis defects. Importantly, our findings are also relevant to the understanding of meiosis. The discovery of gamete-specific differences in SC disassembly timing raises new questions regarding how SC disassembly is linked to the G2 to M transition. Likewise, differences in kinetochore structure raise questions about how kinetochore assembly is modified for distinct chromosome segregation events. This study also establishes a framework for comparative studies. What can be gleaned about the enigmatic karyosome stage from comparative studies between *C. elegans* spermatocytes and the oocytes of *Drosophila* and *Xenopus*? Which features of the rapid spermatogenesis program of *C. elegans* are shared with *Drosophila* and mammals? Until now, studies of *C. elegans* spermatogenesis have focused on features of their non-flagellated spermatozoa; this study highlights the usefulness of *C. elegans* spermatogenesis as a model for understanding the fundamental biology of meiosis.

## Materials and Methods

### Strains

*C. elegans* strains were maintained as described by Brenner [91]. All nematode strains were cultured at 20°C except where noted. Strains used include Bristol N2, CB1489 *him-8(e1489)*, DR466 *him-5(e1490)*, AV307 *syp-1(me17)* V/nT1[*unc-?(n754) let-? qIs50*] (IV;V).

### Synchronization of *C. elegans* populations with the presence of males

Males were obtained either by mating 3 N2 hermaphrodites with 7 N2 males at 19°C for 4 days or by culturing 4–6 *him-8(e1489)* or *him-5(e1490)* hermaphrodites on OP50 seeded NGM plates for 3–5 days. Animals were then collected and bleached to isolate embryos (15 parts double distilled water : 4 parts bleach : 1 part 10N sodium hydroxide). Embryos were hatched without food overnight at 19°C with shaking at 200 rpm. L1 larvae were then plated onto OP50 seeded NGM plates at 19°C for 2–3 days. Animals from these synchronous cultures were used for immunostaining. Alternatively, fourth larval stage (L4) males were collected from mating plates and grown to adulthood for 24–48 hrs prior to analysis.

### Immunohistochemistry and microscopy

Male gonads were dissected in 5–10 microliters of sperm salts on ColorFrost Plus slides (Fisher Scientific) using established protocols for antibody staining of *C. elegans* gonads provided in Wormbook [92]. Three different fixation methods were used in this study. For paraformaldehyde staining, animals were processed as described in [61],[92]. For cold methanol or methanol/acetone fixation, animals were dissected in sperm salts, and then a coverslip with four corner dots of silicon grease was placed over the isolated gonad and gentle pressure was applied to generate partially flattened gonads and/or monolayers of spermatocytes and spermatids. The slide preparation was then placed either in liquid nitrogen or on dry ice. After freezing, the coverslip was removed. For methanol/acetone preparations, the slide was immersed in 95% methanol for 10 minutes followed by a 5 minute immersion in 100% acetone. Slides were allowed to air dry briefly. For −20°C methanol preparations, slides were kept in methanol overnight. Slides were washed with three consecutive 10 minute washes in PBS followed by a 30 min. room temperature incubation in blocking solution (PBS+0.5% BSA and 0.1% Tween 20). Primary and secondary antibody incubations were each diluted into blocking solution at conducted at room temperature in a humid chamber.

For DIC/Hoechst preparations, Hoechst 33342 (Sigma-Aldrich) was used at 100 µg/ml.

The following primary antibodies were used in overnight incubations (unless otherwise noted) with different fixation conditions. Commercial sources or labs kindly providing antibodies are also listed.

Paraformaldehyde fixed preparations: 1∶200 mouse anti-REC-8 (Abcam); mouse anti-Nop-1 (yeast fibrillarin mAbD77, Aris lab) used at a 1∶1000 dilution [93], rabbit anti-HIM-3 (Zetka Lab) used at a 1∶400 dilution [44], rabbit anti-CeLamin (Gruenbaum lab) used at a 1∶500 dilution, rabbit anti-AIR-2 (Schumacher lab) used at a 1∶500 dilution, rabbit anti-SPD-2 (O'Connell Lab) used at a 1∶500 dilution, guinea pig anti-SYP-1 (Villeneuve Lab) was preasborbed against homozygote *syp-1(me17)* mutant animals from the strain AV307 and used at a 1∶200 dilution [45].

Methanol-acetone fixed preparations: rabbit anti-HCP-1 used at a 1∶200 dilution , rabbit anti-HCP-3 used at a 1∶200 dilution, and rabbit anti-HCP-4 used at a 1∶200 dilution (Moore lab), rabbit anti-HIM-10 (Meyer lab) used at a 1∶500 dilution [61]. The HCP-2 antibody, used at a 1∶200 dilution, is a rabbit polyclonal raised against the peptide NSVDDNSYCEPPRASSAHD that correspond to amino acids 93–110 of HCP-2.

Cold methanol preparations as described in [94]: 1∶400 rabbit anti-pHisH3-ser10 (Upstate Biotechnology), 1∶100 FITC-conjugated anti-α-tubulin (DM1A; Sigma-Aldrich), 1∶1000 rabbit anti-PLK-1 [55] (Golden Lab). 1∶3 anti-cyclin B (F2F4 monoclonal developed by P. O'Farrell, Developmental Studies Hybridoma Bank). All incubations were 2–3 hrs at room temperature except PLK-1 and cyclin B, which were incubated overnight at 4°C and room temperature, respectively.

Secondary antibodies from Invitrogen include goat anti-rabbit AlexaFluor 488-labeled IgG (used at 1∶100), goat anti-rat AlexaFluor 488-labeled IgG (used at 1∶100) and goat anti-mouse AlexaFluor 488-labeled IgG (used at 1∶100). Affinity purified secondary antibodies from Jackson Immunoresearch Laboratories include goat anti-rabbit TRITC-labeled IgG (used at 1∶100) and goat anti-mouse FITC-labeled IgG (used at 1∶100). DNA was visualized using the DNA dye DAPI at 0.1 µg/ml. Slides were prepared with either VectaShield (Vector Labs) or GelMount (Biomedia Corp.) as a combined mounting and anti-fade media.

Images were acquired via either confocal microscopy using a Leica TCSNT microscope, epifluorescence microscopy using a Zeiss Axiovert200M coupled with deconvolution via Slidebook 4.2 software (Intelligent Imaging Innovations), or DIC and epifluorescence on an Olympus BX60 microscope equipped with a Cooke Sensicam. Images acquired by confocal microscopy include those to visualize fibrillarin, HIM-10, SPE-11, HCP-4, and HCP-3. For deconvolution, images were acquired at 2×2 binning and 0.2 µm step sizes through each gonad and processed using either constrained iterative or nearest neighbors deconvolution. Images obtained via deconvolution include lamin, SYP-1, HIM-3, AIR-2, RNA pol II CTD(ser 2), SPD-2, and HCP-2. Epifluorescence images include pHisH3-ser10, α-tubulin and PLK-1.

## References

[1] Schumacher JM, Golden A, Donovan PJ (1998). AIR-2: An Aurora/Ipl1-related protein kinase associated with chromosomes and midbody microtubules is required for polar body extrusion and cytokinesis in *Caenorhabditis elegans* embryos.. J Cell Biol.

[2] de Carvalho CE, Zaaijer S, Smolikov S, Gu Y, Schumacher JM (2008). LAB-1 antagonizes the Aurora B kinase in *C. elegans*.. Genes Dev.

[3] Jamnongjit M, Hammes SR (2005). Oocyte maturation: the coming of age of a germ cell.. Semin Reprod Med.

[4] Berruti G (2006). Signaling events during male germ cell differentiation: update, 2006.. Front Biosci.

[5] Griswold MD (1998). The central role of Sertoli cells in spermatogenesis.. Semin Cell Dev Biol.

[6] Morelli MA, Cohen PE (2005). Not all germ cells are created equal: aspects of sexual dimorphism in mammalian meiosis.. Reproduction.

[7] Handel MA, Eppig JJ (1998). Sexual dimorphism in the regulation of mammalian meiosis.. Curr Top Dev Biol.

[8] Hunt P, Hassold T (2002). Sex matters in meiosis.. Science.

[9] Baum JS, St George JP, McCall K (2005). Programmed cell death in the germline.. Semin Cell Dev Biol.

[10] Gruzova MN, Parfenov VN (1993). Karyosphere in oogenesis and intranuclear morphogenesis.. Int Rev Cytol.

[11] Bogolyubov D, Parfenov V (2001). Immunogold localization of RNA polymerase II and pre-mRNA splicing factors in Tenebrio molitor oocyte nuclei with special emphasis on karyosphere development.. Tissue Cell.

[12] Varmark H (2004). Functional role of centrosomes in spindle assembly and organization.. J Cell Biochem.

[13] Kelly WG, Aramayo R (2007). Meiotic silencing and the epigenetics of sex.. Chromosome Res.

[14] Lucchesi JC, Kelly WG, Panning B (2005). Chromatin remodeling in dosage compensation.. Annu Rev Genet.

[15] Lewis JD, Song Y, de Jong ME, Bagha SM, Ausio J (2003). A walk though vertebrate and invertebrate protamines.. Chromosoma.

[16] Braun RE (2001). Packaging paternal chromosomes with protamine.. Nat Genet.

[17] Chu DS, Liu H, Nix P, Wu TF, Ralston EJ (2006). Sperm chromatin proteomics identifies evolutionarily conserved fertility factors.. Nature.

[18] Colaiacovo MP (2006). The many facets of SC function during *C. elegans* meiosis.. Chromosoma.

[19] Zetka M, Rose A (1995). The genetics of meiosis in *Caenorhabditis elegans*.. Trends Genet.

[20] Hubbard EJ, Greenstein D (2000). The *Caenorhabditis elegans* gonad: a test tube for cell and developmental biology.. Dev Dyn.

[21] Riddle DL, Blumenthal T, Meyer BJ, Priess JR (1997). *C. elegans* II..

[22] Chan RC, Severson AF, Meyer BJ (2004). Condensin restructures chromosomes in preparation for meiotic divisions.. J Cell Biol.

[23] Martinez-Perez E, Schvarzstein M, Barroso C, Lightfoot J, Dernburg AF (2008). Crossovers trigger a remodeling of meiotic chromosome axis composition that is linked to two-step loss of sister chromatid cohesion.. Genes Dev.

[24] Nabeshima K, Villeneuve AM, Colaiacovo MP (2005). Crossing over is coupled to late meiotic prophase bivalent differentiation through asymmetric disassembly of the SC.. J Cell Biol.

[25] McCarter J, Bartlett B, Dang T, Schedl T (1999). On the control of oocyte meiotic maturation and ovulation in *Caenorhabditis elegans*.. Dev Biol.

[26] McNally KL, McNally FJ (2005). Fertilization initiates the transition from anaphase I to metaphase II during female meiosis in *C. elegans*.. Dev Biol.

[27] Greenstein D (2005). Control of oocyte meiotic maturation and fertilization.. WormBook.

[28] Burrows AE, Sceurman BK, Kosinski ME, Richie CT, Sadler PL (2006). The *C. elegans* Myt1 ortholog is required for the proper timing of oocyte maturation.. Development.

[29] L'Hernault SW, Benian GM, Emmons RB (1993). Genetic and molecular characterization of the *Caenorhabditis elegans* spermatogenesis-defective gene *spe-17.*. Genetics.

[30] Ward S, Argon Y, Nelson GA (1981). Sperm morphogenesis in wild-type and fertilization-defective mutants of *Caenorhabditis elegans*.. J Cell Biol.

[31] Kimble J, Crittenden SL (2007). Control of Germline Stem Cells, Entry into Meiosis, and the Sperm/Oocyte Decision in *C. elegans*.. Annu Rev Cell Dev Biol.

[32] Kimble JE, White JG (1981). On the control of germ cell development in *Caenorhabditis elegans*.. Dev Biol.

[33] Gumienny TL, Lambie E, Hartwieg E, Horvitz HR, Hengartner MO (1999). Genetic control of programmed cell death in the *Caenorhabditis elegans* hermaphrodite germline.. Development.

[34] Wolke U, Jezuit EA, Priess JR (2007). Actin-dependent cytoplasmic streaming in *C. elegans* oogenesis.. Development.

[35] Maddox AS, Habermann B, Desai A, Oegema K (2005). Distinct roles for two *C. elegans* anillins in the gonad and early embryo.. Development.

[36] White JG, Wood WB (1988). The anatomy.

[37] Kelly WG, Schaner CE, Dernburg AF, Lee M-H, Kim SK (2002). X chromosome silencing in the germline of *C. elegans*.. Development.

[38] Komarnitsky P, Cho EJ, Buratowski S (2000). Different phosphorylated forms of RNA polymerase II and associated mRNA processing factors during transcription.. Genes Dev.

[39] Yang HY, McNally K, McNally FJ (2003). MEI-1/katanin is required for translocation of the meiosis I spindle to the oocyte cortex in *C. elegans*.. Dev Biol.

[40] Orr-Weaver TL, Parfenov VN, Dudina LM, Kostiuchek DF, Gruzova MN (1995). Meiosis in *Drosophila*: seeing is believing.. Proc Natl Acad Sci U S A.

[41] Sanyal MK, Taymor ML, Berger MJ (1976). Cytologic features of oocytes in the adult human ovary.. Fertil Steril.

[42] Hsu JY, Sun ZW, Li X, Reuben M, Tatchell K (2000). Mitotic phosphorylation of histone H3 is governed by Ipl1/aurora kinase and Glc7/PP1 phosphatase in budding yeast and nematodes.. Cell.

[43] Page SL, Hawley RS (2004). The genetics and molecular biology of the synaptonemal complex.. Annu Rev Cell Dev Biol.

[44] Zetka M, Kawasaki I, Strome S, Muller F (1999). Synapsis and chiasma formation in *Caenorhabditis elegans* require HIM-3, a a meiotic chromosome core component that functions in chromosome segregation.. Genes Dev.

[45] MacQueen AJ, Villeneuve AM (2001). Nuclear reorganization and homologous chromosome pairing during meiotic.. Genes Dev.

[46] Couteau F, Nabeshima K, Villeneuve A, Zetka M (2004). A component of *C. elegans* meiotic chromosome axes at the interface of homolog alignment, synapsis, nuclear reorganization, and recombination.. Curr Biol.

[47] Bischoff JR, Plowman GD (1999). The Aurora/Ipl1p kinase family: regulators of chromosome segregation and cytokinesis.. Trends Cell Biol.

[48] Rogers E, Bishop JD, Waddle JA, Schumacher JM, Lin R (2002). The aurora kinase AIR-2 functions in the release of chromosome cohesion in *Caenorhabditis elegans* meiosis.. J Cell Biol.

[49] Miller MA, Nguyen VQ, Lee MH, Kosinski M, Schedl T (2001). A sperm cytoskeletal protein that signals oocyte meiotic maturation and ovulation.. Science.

[50] Sun F, Handel MA (2008). Regulation of the meiotic prophase I to metaphase I transition in mouse spermatocytes.. Chromosoma.

[51] Schatten G (1994). The centrosome and its mode of inheritance: the reduction of the centrosome during gametogenesis and its restoration during fertilization.. Dev Biol.

[52] Kemp CA, Kopish KR, Zipperlen P, Ahringer J, O'Connell KF (2004). Centrosome maturation and duplication in *C. elegans* require the coiled-coil protein SPD-2.. Dev Cell.

[53] Kim DY, Roy R (2006). Cell cycle regulators control centrosome elimination during oogenesis in *Caenorhabditis elegans*.. J Cell Biol.

[54] Pelletier L, Ozlu N, Hannak E, Cowan C, Habermann B (2004). The *Caenorhabditis elegans* centrosomal protein SPD-2 is required for both pericentriolar material recruitment and centriole duplication.. Curr Biol.

[55] Chase D, Serafinas C, Ashcroft N, Kosinski M, Longo D (2000). The polo-like kinase PLK-1 is required for nuclear envelope breakdown and the completion of meiosis in *Caenorhabditis elegans*.. Genesis.

[56] van Vugt MA, Medema RH (2005). Getting in and out of mitosis with Polo-like kinase-1.. Oncogene.

[57] Xiang Y, Takeo S, Florens L, Hughes SE, Huo LJ (2007). The inhibition of polo kinase by matrimony maintains G2 arrest in the meiotic cell cycle.. PLoS Biol.

[58] Casenghi M, Meraldi P, Weinhart U, Duncan PI, Korner R (2003). Polo-like kinase 1 regulates Nlp, a centrosome protein involved in microtubule nucleation.. Dev Cell.

[59] Lane HA, Nigg EA (1996). Antibody microinjection reveals an essential role for human polo-like kinase 1 (Plk1) in the functional maturation of mitotic centrosomes.. J Cell Biol.

[60] Albertson DG, Thomson JN (1982). The kinetochores of *Caenorhabditis elegans*.. Chromosoma.

[61] Howe M, McDonald KL, Albertson DG, Meyer BJ (2001). HIM-10 is required for kinetochore structure and function on *Caenorhabditis elegans* holocentric chromosomes.. J Cell Biol.

[62] Cheeseman IM, Desai A (2008). Molecular architecture of the kinetochore-microtubule interface.. Nat Rev Mol Cell Biol.

[63] Maddox PS, Oegema K, Desai A, Cheeseman IM (2004). “Holo”er than thou: chromosome segregation and kinetochore function in *C. elegans*.. Chromosome Res.

[64] Cheeseman IM, MacLeod I, Yates JR, Oegema K, Desai A (2005). The CENP-F-like proteins HCP-1 and HCP-2 target CLASP to kinetochores to mediate chromosome segregation.. Curr Biol.

[65] Tarailo M, Kitagawa R, Rose AM (2007). Suppressors of spindle checkpoint defect (such) mutants identify new mdf-1/MAD1 interactors in *Caenorhabditis elegans*.. Genetics.

[66] Hajeri VA, Stewart AM, Moore LL, Padilla PA (2008). Genetic analysis of the spindle checkpoint genes *san-1, mdf-2, bub-3* and the CENP-F homologues *hcp-1* and *hcp-2* in *Caenorhabditis elegans*.. Cell Div.

[67] Albertson DG, Thomson JN (1993). Segregation of holocentric chromosomes at meiosis in the nematode, *Caenorhabditis elegans*.. Chromosome Res.

[68] Wignall SM, Villeneuve AM (2009). Lateral microtubule bundles promote chromosome alignment during acentrosomal oocyte meiosis.. Nat Cell Biol.

[69] Ward S, Gall J (1986). The asymmetric localization of gene products during the development of *Caenorhabditis elegans* spermatozoa.. Gametogenesis and the Early Embryo.

[70] Pasierbek P, Jantsch M, Melcher M, Schleiffer A, Schweizer D (2001). A *Caenorhabditis elegans* cohesion protein with functions in meiotic chromosome pairing and disjunction.. Genes Dev.

[71] Kaitna S, Pasierbek P, Jantsch M, Loidl J, Glotzer M (2002). The aurora B kinase AIR 2 regulates kinetochores during mitosis and is required for separation of homologous chromosomes during meiosis.. Curr Biol.

[72] Sumara I, Vorlaufer E, Stukenberg PT, Kelm O, Redemann N (2002). The dissociation of cohesin from chromosomes in prophase is regulated by Polo-like kinase.. Mol Cell.

[73] Barr FA, Sillje HH, Nigg EA (2004). Polo-like kinases and the orchestration of cell division.. Nat Rev Mol Cell Biol.

[74] Jaramillo-Lambert A, Ellefson M, Villeneuve AM, Engebrecht J (2007). Differential timing of S phases, X chromosome replication, and meiotic prophase in the *C. elegans* germ line.. Dev Biol.

[75] Gartner A, Milstein S, Ahmed S, Hodgkin J, Hengartner MO (2000). A conserved checkpoint pathway mediates DNA damage–induced apoptosis and cell cycle arrest in *C. elegans*.. Mol Cell.

[76] O'Connell CB, Khodjakov AL (2007). Cooperative mechanisms of mitotic spindle formation.. J Cell Sci.

[77] Doubilet S, McKim KS (2007). Spindle assembly in the oocytes of mouse and *Drosophila*–similar solutions to a problem.. Chromosome Res.

[78] Walczak CE, Heald R (2008). Mechanisms of mitotic spindle assembly and function.. Int Rev Cytol.

[79] Lewis JD, Abbott DW, Ausio J (2003). A haploid affair: core histone transitions during spermatogenesis.. Biochem Cell Biol.

[80] Sassone-Corsi P (2002). Unique chromatin remodeling and transcriptional regulation in spermatogenesis.. Science.

[81] L'Hernault SW (2006). Spermatogenesis.. WormBook.

[82] Lamitina ST, L'Hernault SW (2002). Dominant mutations in the *Caenorhabditis elegans* Myt1 ortholog *wee-1.3* reveal a novel domain that controls M-phase entry during spermatogenesis.. Development.

[83] Ivanovska I, Khandan T, Ito T, Orr-Weaver TL (2005). A histone code in meiosis: the histone kinase, NHK-1, is required for proper chromosomal architecture in Drosophila oocytes.. Genes Dev.

[84] Parfenov VN, Dudina LM, Kostiuchek DF, Gruzova MN, Parfenov V (1984). Nuclear ultrastructure of the oocytes from human antral follicles. Formation of the karyosphere.. Tsitologiia.

[85] Kang TH, Park DY, Choi YH, Kim KJ, Yoon HS (2007). Mitotic histone H3 phosphorylation by vaccinia-related kinase 1 in mammalian cells.. Mol Cell Biol.

[86] Gorjanacz M, Klerkx EP, Galy V, Santarella R, Lopez-Iglesias C (2007). *Caenorhabditis elegans* BAF-1 and its kinase VRK-1 participate directly in post-mitotic nuclear envelope assembly.. Embo J.

[87] Hall DH, Winfrey VP, Blaeuer G, Hoffman LH, Furuta T (1999). Ultrastructural features of the adult hermaphrodite gonad of *Caenorhabditis elegans*: relations between the germ line and soma.. Dev Biol.

[88] Monen J, Maddox PS, Hyndman F, Oegema K, Desai A (2005). Differential role of CENP-A in the segregation of holocentric *C. elegans* chromosomes during meiosis and mitosis.. Nat Cell Biol.

[89] Fernandez-Capetillo O, Mahadevaiah SK, Celeste A, Romanienko PJ, Camerini-Otero RD (2003). H2AX is required for chromatin remodeling and inactivation of sex chromosomes in male mouse meiosis.. Dev Cell.

[90] Lewis JD, Ausio J (2002). Protamine-like proteins: evidence for a novel chromatin structure.. Biochem Cell Biol.

[91] Brenner S (1974). The genetics of *Caenorhabditis elegans*.. Genetics.

[92] Shaham S (2006). Methods in Cell Biology.

[93] Aris JP, Blobel G (1988). Identification and characterization of a yeast nucleolar protein that is similar to a rat liver nucleolar protein.. J Cell Biol.

[94] Golden A, Sadler PL, Wallenfang MR, Schumacher JM, Hamill DR (2000). Metaphase to anaphase (mat) transition-defective mutants in *Caenorhabditis elegans*.. J Cell Biol.

